# Human retinal Müller cells synthesize collagens of the vitreous and vitreoretinal interface in vitro

**Published:** 2008-03-26

**Authors:** Theodorus L. Ponsioen, Marja J.A. van Luyn, Roelofje J. van der Worp, Hendri H. Pas, Johanna M.M. Hooymans, Leonoor I. Los

**Affiliations:** 1 University Medical Center Groningen and University of Groningen, Department of Ophthalmology, Groningen, The Netherlands; 2 University of Groningen and University Medical Center Groningen, Department of Pathology and Medical Biology, Groningen, The Netherlands; 3 University Medical Center Groningen and University of Groningen, Department of Dermatology, Center for Blistering Diseases, Groningen, The Netherlands

## Abstract

**Purpose:**

To investigate the capacity of cultured Müller cells to synthesize collagens, since previous studies indicated that Müller cells could be involved in collagen remodeling at the vitreoretinal border in adult human eyes.

**Methods:**

Spontaneously immortalized cultured human Müller cells were analyzed for the presence of mRNA of types I-VII, IX, XI, and XVII collagen by RT–PCR. Furthermore, Müller cells were immunocytochemically stained for light microscopic (LM) evaluation of these collagens and their main characteristics. Finally, cell extracts and culture medium were evaluated by western blot (WB) analysis using anticollagen antibodies.

**Results:**

Cultured Müller cells contained mRNA for types I-VII, IX, and XI collagen, but not for type XVII collagen. LM and WB confirmed the intracellular expression of all the above-mentioned collagens with the exception of type XVII. Collagen secretion into the medium was established for types I-VII, IX, and XI collagen.

**Conclusions:**

Cultured Müller cells can synthesize internal limiting lamina and vitreous collagens. Possible collagen production by Müller cells could explain and expand on previous in vivo morphological findings in the embryonic and postnatal period and in pathologic conditions.

## Introduction

Müller cells are radially oriented macroglia that traverse the retina from its inner (vitreal) border to the outer limiting membrane. These cells have many local functions: they stabilize the retinal architecture, provide an orientation scaffold, give structural and metabolic support to retinal neurons and blood vessels, and prevent aberrant photoreceptor migration into the subretinal space [1,2]. In vivo and in vitro, Müller cells can produce and express several cytokines, growth factors, and receptors [2]. Other features of Müller cells are the expression of cellular retinaldehyde binding protein (CRALBP), vimentin, and, on activation, glial fibrillary acidic protein (GFAP) [3,4]. A possible role in the production of vitreous macromolecules during growth and in adulthood has been suggested [5,6], but little is known about their capability to produce vitreous and internal limiting lamina (ILL) collagens postnatally. Whether Müller cells are capable of producing basement membrane components is a matter of debate. Some studies find evidence hereof [7-12], while others fail to confirm it [13-15].

Recently, turnover and remodeling of vitreous collagen was described in human donor eyes. Evidence for collagen breakdown in matrix areas bordering liquefied spaces was found in the human vitreous [16]. In addition to collagen breakdown, a study on vitreous collagens and two studies on the vitreoretinal interface found evidence of postnatal collagen synthesis in the human eye. The first detected type II procollagens in the vitreous [17] and the latter two described intraretinal fibers and isolated packages of vitreous collagen (type II) [18,19]. On aging, intraretinal collagen fibers expanded under the ILL at the vitreous base into networks and made contact with the basal vitreous, leading to the formation of vitreoretinal collagen connections [18]. We observed comparable intraretinal packages of vitreous collagen, often associated with surrounding Müller cell processes, focal interruptions of the ILL, and the presence of macrophages and cell debris. These findings could be consistent with a process of interactive remodeling with a net synthesis of vitreous collagens. Because of their close proximity to collagen packages, Müller cells may be involved in this process of matrix remodeling [19].

The present study evaluates the in vitro capacity of the human Müller cell line, MIO-M1 [4], to synthesize (1) known vitreous collagens (i.e., types II, V/XI, VI, and IX) [20], (2) ILL collagens (types IV and VI; unpublished data of our group) [19,21], (3) type VII collagen [22] (which appears to be present in the human retina by immunohistochemical staining; unpublished data of our group), (4) collagens described in epiretinal and vitreoretinal membranes (types I-V) [23,24], and (5) a collagen not related to the vitreoretinal interface (the hemidesmosomal transmembrane type XVII collagen found in basement membranes of stratified and pseudostratified epithelia) [25]. In this in vitro model, we demonstrate the capacity of Müller cells for collagen production, cytoplasmic expression of collagens, and their secretion into the cell medium.

## Methods

### Culture of cells

The spontaneously immortalized human Müller cell line MIO-M1 (a kind gift of G.A. Limb, Moorfields/Institute of Ophthalmology, London, UK) has all the characteristics of human retinal Müller cells [4]. The cells were cultured to confluence in Dulbecco’s modification of Eagle’s medium (DMEM) high glucose containing L-glutamax I (Life Technologies Inc., Rockville, MD), 10% fetal bovine serum (FBS; Life Technologies Inc.) and 1% penicillin/streptomycin.

For western blot (WB) analyses of the supernatant, Müller cells were cultured in DMEM high glucose containing L-glutamax I without FBS and supplemented with 1% G5 (Life Technologies Inc.), 0.2 mM β-aminopropionitrile fumurate salt (β-APN; Sigma, St. Louis, MO), 0.2 mM ascorbic acid (Sigma), and 1% penicillin/streptomycin, since 10% FBS caused clotting of the medium after our concentration procedure. The serum-free medium with supplements was introduced after 24 h to allow uniform attachment of the Müller cells. Ascorbic acid promotes the intracellular hydroxylation of prolyl and lysyl residues during collagen synthesis [26], whereas β-APN inhibits the enzyme lysyl oxidase in the extracellular space thus preventing collagen cross-link formation [27].

**Table 1:** Overview of all primers used in the RT–PCR analysis

**Collagen mRNA**	**Forward primer 5′→3′**	**Reverse primer 5′→3′**	**Size** **(bp)**
*COL1A1*	TCG GCG AGA GCA TGA CCG ATG GAT	GAC GCT GTA GGT GAA GCG GCT GTT	254
*COL2A1*	GTG GAA GAG TGG AGA CTA CTG	TGT ACG TGA ACC TGC TAT TG	419
*COL3A1*	ACC GAT GAG ATT ATG ACT TCA CT	CTG CAC ATC AAG GAC ATC TTC AG	369
*COL4A2*	ATC GGC TAC CTC CTG GTG AA	GCT GAT GTG TGT GCG GAT GA	648
*COL5A1*	GAC TAC GCG GAC GGC ATG GAA	CCT GCC AGG CCA CTG ACT GGT A	454
*COL6A1*	GGA GCT CAA GGA AGC CAT CAA G	TCC TCC AGC AGC TCT GCA TAG T	342
*COL7A1*	CCG AGG ACG AGA TGG TGA AGT TG	CTG GCT CCA GGT CCT GTG TCT AC	261
*COL9A1*	GCC TCT GGT GAA GAA GGT GAA	TGC TGA TCT GTC GGT GCT CTA	245
*COL11A1*	CAG CAG GCT CGG ATT GCT CTG A	GGC CAT CTA CAC CTG CCA TAC C	460
*COL17A1*	ATG GAG CTG CTC ATC ATG AC	AGG AGT AGC AGC CAG GTG AG	364

*COL1A1* indicates mRNA of the α1 chain of type I collagen.

### Reverse transcriptase-polymerase chain reaction

Total RNA from the Müller cells was extracted by RNeasy Mini kit method (Qiagen, Venlo, the Netherlands) according to the manufacturer’s instructions. To eliminate DNA contamination, we treated RNA samples with DNase treatment Ambion-kit (DNA-free). RNA concentration and purity were determined on a spectrophotometer (Nanodrop, Isogen, Maarssen, the Netherlands) by calculating the ratio of optical density at wavelengths of 260 and 280 nm. Two μg RNA was reverse transcribed into cDNA using M-MuLV reverse transcriptase (MBI Fermentas, St. Leon-Rot, Germany) according to manufacturer’s protocol for a total reaction of 20 μl.

For the PCR reaction, 1 μl cDNA was added to 23 μl “master mix” consisting of 2.5 μl 10×PCR buffer, 2.5 μl 2 mM dNTP mix, 1.5 μl 25 mM MgCl_2_, 0.25 μl (5 U/μl) Taq DNA polymerase (Fermentas) and 16.25 μl milli-Q water. Finally, a total of 1 μl of the two specific flanking primers (50 μM) was added (Table 1). The mixtures were initially denatured at 94 °C for 5 min. The PCR consisted of 35 cycles at the following conditions: denaturation at 94 °C for 0.5 min, annealing at 55 °C (for types I, II, III, V, and IX collagen) and at 58 °C (for types IV, VI, VII, XI, and XVII collagen) for 1 min, and an extension period at 72 °C for 1 min. These cycles were followed by a final extension period at 72 °C of 10 min. PCR products were analyzed by agarose gel electrophoresis (1%) with 500 ng/ml ethidium bromide. Keratinocytes were used as positive control for type XVII collagen.

### Immunocytochemistry

By light microscopy (LM), Müller cells were identified by their morphology and by their expression of CRALBP, vimentin, and GFAP. The expression of cellular characteristics was measured in at least three microscopic areas at a magnification of 10 times. To determine the intracellular expression of collagens, we specifically stained Müller cells with antibodies against human types I-VII, IX, XI, and XVII collagen.

For immunocytochemical staining, cells were seeded for 48 h in glass chamber slides. After fixation with 1:1 acetone/methanol for 10 min at −20 °C, the slides were washed with phosphate buffered saline (PBS) and pre-incubated for 30 min with 3% serum of the producer of the secondary antibody in PBS with 2% BSA (BSA) (Sanquin, Amsterdam, the Netherlands), followed by incubation for 1 h with primary antibodies diluted 1:50 in PBS with 1% BSA. In the case of types VI, VII, IX, and XI collagen, the latter step was preceded by blocking steps with avidin and biotin. The primary antibodies included the following: rabbit polyclonal antibodies against CRALBP (UW55, a kind gift from J.C. Saari, University of Washington, Seattle, WA) and human types I, III, V (Abcam, Cambridge, UK), and XI collagen (a kind gift from J. Oxford, Boise State University, Boise, Idaho); a biotinylated rabbit polyclonal antibody against human type VI collagen (Abcam); goat polyclonal antihuman antibodies against types II and IV collagen (Southern Biotechnology Associates, Birmingham, AL); and mouse monoclonal antihuman antibodies against vimentin (DAKO, Glostrup, Denmark), GFAP (Sigma), and types VII (Abcam), IX (USBiological, Swampscott, MA) and XVII collagen (1A8C [28]; a kind gift from K. Owaribe, Nagoya University, Japan). Subsequently, cells were washed with PBS and endogenous peroxidases were blocked. Secondary antibodies included a swine-antirabbit peroxidase (DAKO), a biotinylated goat-antirabbit (GARbio, DAKO), a rabbit-antigoat peroxidase (DAKO), a rabbit-antimouse peroxidase (DAKO), a biotinylated goat-antimouse IgG1 (GAMbio, SBA), and a biotinylated goat-antimouse IgG2a (GAMbio, SBA). Secondary antibodies were diluted 1:100 with PBS containing 2% human serum was derived from a pool of human volunteers and added for 1 h at room temperature. Biotinylated type VI collagen, GARbio, and GAMbio (IgG1 and IgG2) were followed by incubation with ABC complex horse radish peroxidase (DAKO) for 20 min. Finally, cells were stained with 3-amino-9-ethylcarbazole (AEC; Sigma) and hematoxylin. Negative controls underwent the entire procedure, except for the substitution of the primary antibody.

**Figure 1:**
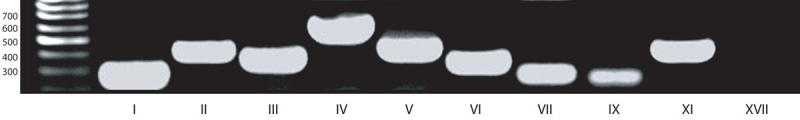
RT–PCR on Müller cell extracts. From left to right, bands indicating the positions of types I, II, III, IV, V, VI, VII, IX, XI, and XVII collagen are depicted. At the left margin, a 100 bp DNA ladder has been added.

### Western blot

#### Isolation of cell extract and supernatant concentration

Cells were harvested in sodium dodecyl sulfate PAGE (SDS–PAGE) denaturation buffer (10 mM Tris-HCl, pH 7 containing 1 mM EDTA, 2.5% SDS, 5% 2-mercaptoethanol, and 10% glycerol). The extract was heated for 5 min at 100 °C to unfold the collagen helices into separate α-chains.

After dead cells and cellular debris were removed by centrifuging at 1,600 rpm for 5 min, the supernatant was concentrated by ultrafiltration with an Amicon membrane (100,000 kDa cut-off; Millipore, Billerica, MA) and, in the case of collagen XI, with a Vivaspin 0.5 ml concentrator (30,000 kDa cut-off; Vivascience, Hanover, Germany).

**Figure 2:**
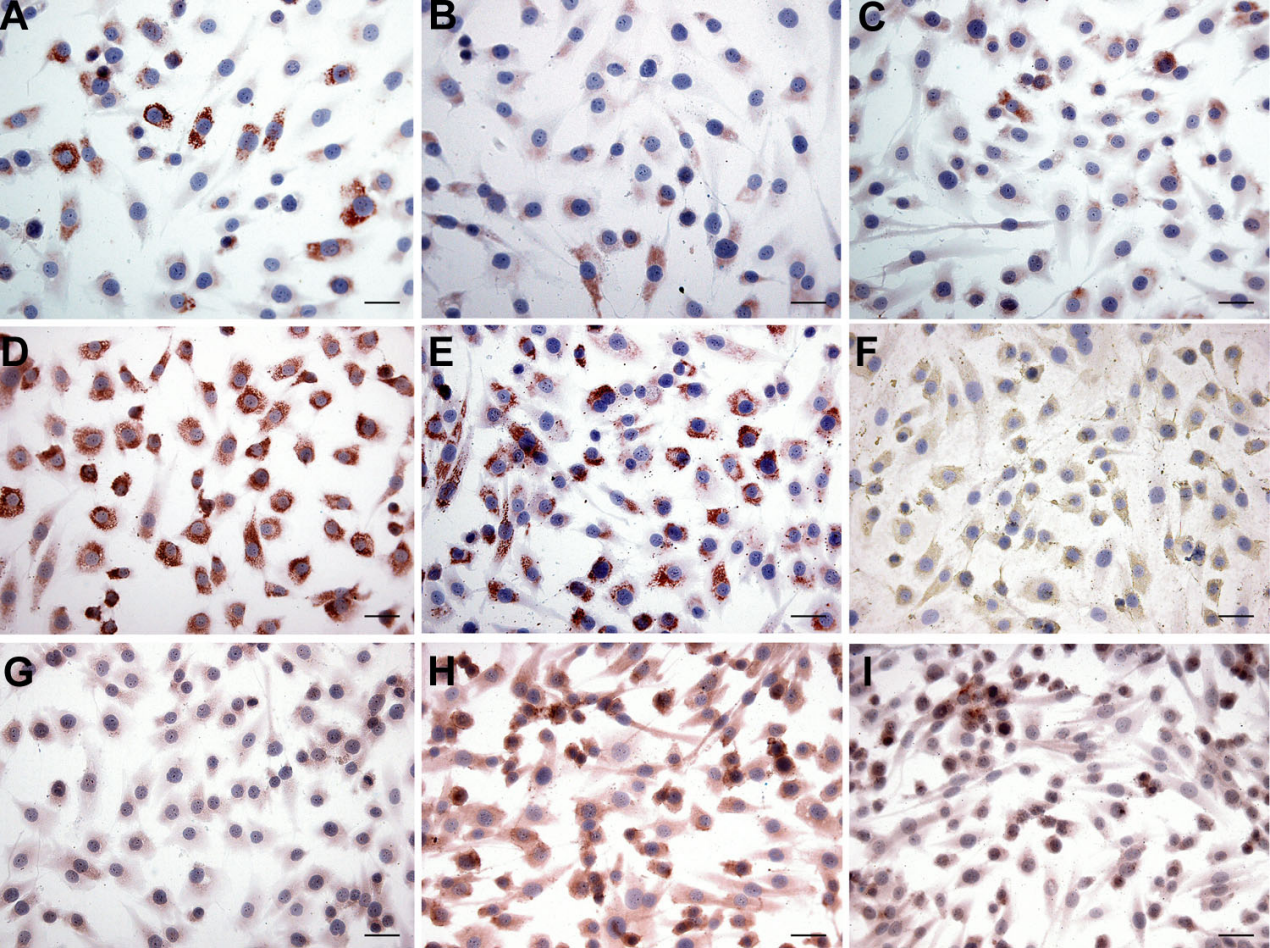
Immunocytochemical analyses of cultured Müller cells in medium with fetal bovine serum. **A**: Type I collagen shows a granular staining with a variable intensity between cells. **B**: Type II collagen is seen as a faint staining in the cytoplasm. **C**: Type III collagen is positive in all cells. **D**: Type IV collagen is visible as a strong granular cytoplasmic staining. **E**: Type V collagen shows mainly staining in the cytoplasm. **F**: In the case of type VI collagen, the cells are predominantly stained in the cytoplasm. **G**: Type VII collagen is faintly positive in the cytoplasm. **H**: Type IX collagen is also present in the cytoplasm. **I**: Type XI collagen is primarily seen in the cytoplasm. Bars in all panels equal 50 μm.

#### Immunoblotting procedure

Polyacrylamide SDS electrophoresis was performed according to the method of Laemmli [29], using 3.9% and 5% slab gels and a 72-mm wide 2D gel comb in the Bio-Rad Mini Protean II electrophoresis apparatus (Bio-Rad, Hercules, CA). After separation, the gel was blotted to nitrocellulose using the Mini Protean II blotting unit (Bio-Rad) with 22 mM Tris, 168 mM Glycine, 0.05% SDS, and 20% methanol as a transfer buffer. After the transfer, the nitrocellulose was blocked for 1 h in TBS-buffer (20 mM Tris-HCl and 500 mM NaCl; pH 7.5) containing 3% BSA. Primary antibodies (Table 2) were diluted 1:500 in TBS and added to the blot. After incubation overnight, the blot was washed with TBS containing 0.05% Tween-20 (TTBS), and then secondary antibodies (Table 2) diluted 1:500 in TTBS were added. After 1 h incubation, the blot was washed with TTBS and incubated with alkaline phosphatase (AP)-conjugated tertiary antibody (Table 2) diluted 1:250 in TTBS for another hour. After washing with TTBS and AP buffer (100 mM Tris-HCl, 100 mM NaCl, and 5 mM MgCl_2_, pH 9.5), the blot was developed with nitro blue tetrazolium and 5-bromo-4-chloro-3-indolyl phosphate in AP buffer. All incubation and washing steps were performed at room temperature.

**Table 2:** Overview of the western blot results

**Collagen**	**Molecular weight of collagen bands in Müller cells**	**Moleculr weight of collagen bands in culture medium**	**Molecular weight of collagen bands after type VII collagenase**
Type I	140 kDa	140 kDa	None
Type II	150 kDa	150 kDa	None
Type III	180 kDa	180 kDa	None
Type IV	130 and 210 kDa	130 and 210 kDa	75, 90, 110, and 250 kDa
Type V	260 kDa	260 kDa	None
Type VI	120 and 230 kDa	120 and 230 kDa	150 kDa
Type VII	270 kDa	270 kDa	130, 135, and 140 kDa
Type IX	200 kDa	150 and 200 kDa	None
Type XI	100, 150, and 200 kDa	150 kDa	None
Type XVII	None	None	None

By SDS–PAGE, collagen secretion into the medium was established for types I-VII, IX, and XI collagen. The specific collagen antibodies detected the collagen bands (in kDa) obtained before and after treatment with type VII collagenase. The results of both Müller cell extracts and medium with G5 are shown. The results after type VII collagenase concern Müller cell extracts and confirm the collagen nature of the bands as seen by WB. “None” indicates no collagen bands were detected.

#### Collagenase digestion

Cell extracts were mixed with CaCl_2_ to a final concentration of 10 mM to inactivate EDTA. Type VII collagenase (high purity grade, Sigma) was added in increasing concentrations (0–30 units/ml) to 60 μl cell extract and incubated for 1 h at 37 °C. Previously, the absence of nonspecific proteases in this collagenase batch had been confirmed [30]. To confirm that type VII collagenase specifically cleaves collagen, we exposed Müller cell extracts to this collagenase, after which vimentin and CRALBP expressions were checked. After incubation, samples were mixed with SDS–PAGE sample buffer to inactivate collagenase activity.

**Figure 3:**
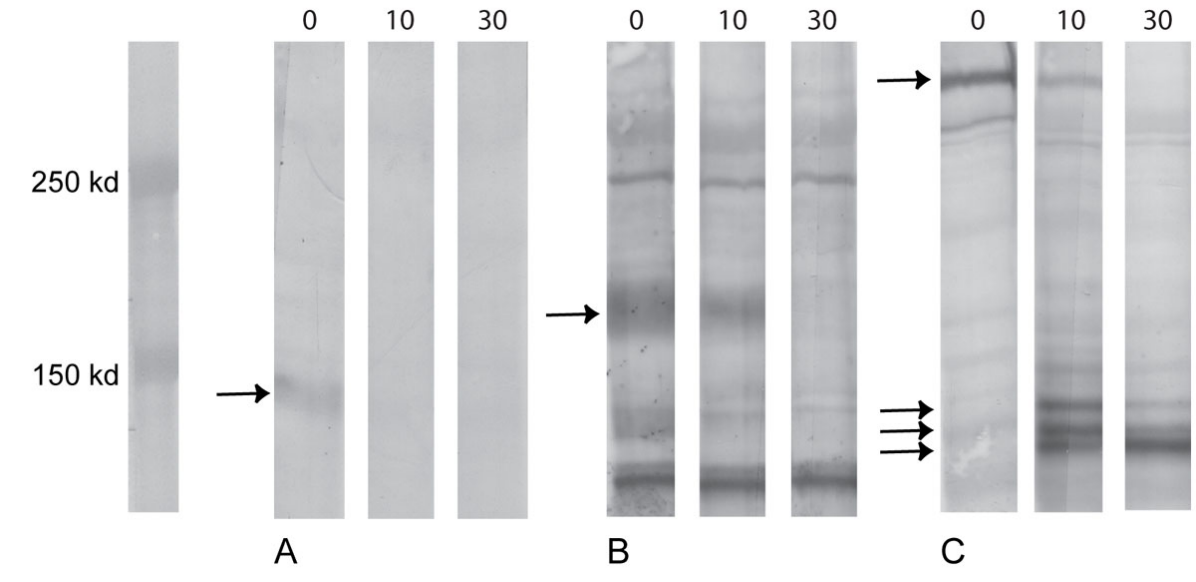
Examples of western blot analyses: Müller cell extracts with the addition of type VII collagenase (0, 10, and 30 units/ml). **A:** The specific band of type II collagen is shown at 150 kDa and disappears without formation of new bands when type VII collagenase is added. **B:** At 180 kDa, the specific band of type III collagen disappears gradually on the addition of type VII collagenase. **C:** Addition of type VII collagenase to type VII collagen results in the gradual disappearance of the specific band at 270 kDa and the appearance of breakdown products at 130, 135, and 140 kDa. Specific bands are indicated with arrows.

#### Cell viability

To determine cell viability, Müller cells and their medium supplemented with 1% G5, 0.2 mM β-APN, and 0.2 mM ascorbic acid were harvested and compared to a well with 10% FBS, which served as a reference, after an incubation period of 48 h. Dead cells were stained with trypan blue, counted in a Bürker-Türker counter (W. Schreck, Hofheim, Germany), and compared to viable cells.

## Results

### Reverse transcriptase polymerase chain reaction

Müller cells expressed mRNA of all tested collagen types, except for type XVII collagen (Figure 1). Amplimers were seen at the expected positions, which are for *COL1A1* at 254 bps, for *COL2A1* at 419 bps, for *COL3A1* at 369 bps, for *COL4A2* at 648 bps, for *COL5A1* at 454 bps, for *COL6A1* at 342 bps, for *COL7A1* at 261 bps, for *COL9A1* at 245 bps, and for *COL11A1* at 460 bps. *COL17A1* gave no product band as expected. Keratinocytes contained *COL17A1* (figure not shown).

### Immunocytochemistry

LM results revealed that Müller cells preserved their morphology and characteristics under the culture method with FBS. They were all positive for vimentin and CRALBP, and less than 5% of the cells were GFAP positive (pictures not shown). In the case of collagen staining, the cytoplasm of all Müller cells was positive for all collagens with the exception of type XVII collagen (Table 3; Figures 2A-I). The pattern of the cytoplasmic staining varied in intensity and had a granular to fibrillary aspect. Types V, VI, and XI collagen also stained positively outside the cell—e.g., as small granules and fibers. All negative controls showed no staining (not shown).

### Western blot

Cell extracts of Müller cells were immunoblotted for the presence of types I-VII, IX, XI, and XVII collagen and showed specific collagen bands (Table 2; Figures 3A-C), which disappeared on treatment with increasing doses of type VII collagenase, except for type XVII collagen which showed no band. In the case of types IV, VI, and VII collagens (Figure 3C), new product bands were detected after collagenase treatment. The collagenase had no effect on the bands of CRALBP and vimentin (not shown).

**Figure 4:**
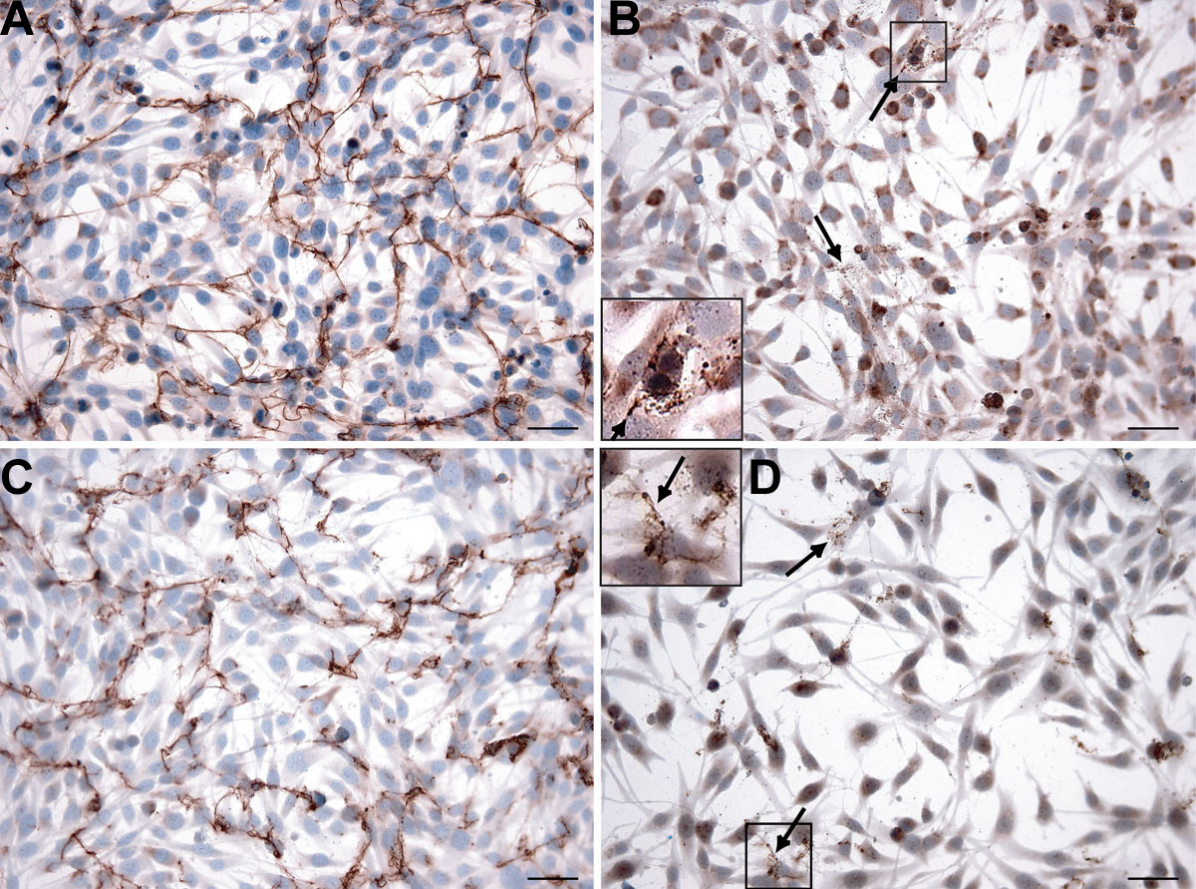
Immunocytochemical analyses of cultured Müller cells in medium with G5. Types I (**A**) and V (**C**) collagen show clear extracellular fibrillar threads and less intracellular staining compared to Figures 2A and 2E, respectively. Types II (**B**) and XI (**D**) collagen show some fine extracellular threads and small granules (arrows) and decreased intracellular staining compared to Figures 2B and 2I, respectively. In the inlays of Figures 4B and 4D, the extracellular collagen is magnified two times. Bars in each panel equal 50 μm.

### Analysis of conditioned growth medium with G5

Without FBS but with G5, Müller cells preserved their immunocytochemical characteristics, although, morphologically, they appeared a little stretched. Cell viability remained above 95% with a slightly diminished proliferation rate compared to conditions with 10% FBS. In comparison with cells grown in the medium with FBS, the RT–PCR results were similar. LM results were comparable but showed less intense intracellular and more extracellular staining for types I, II, V, and XI collagen. Types I and V collagen were visible outside the cell as fibrillar threads and types II and XI collagen also, but to a lesser extent and they had a more granular aspect (Figures 4A-D). In the medium, we found specific collagen bands (Table 2) similar to those detected in the cell extracts except for type IX collagen where in addition to the 200 kDa band a weak band at 150 kDa was present. For type XI collagen, only the 150 kDa band was detected in the medium.

**Table 3:** Immunocytochemical analysis of human retinal Müller cells

Collagen	Cytoplasm staining		Extracellular staining		Cytoplasm aspect	
FBS	G5	FBS	G5	FBS	G5
Type I	++	+	n.d.	++	Granular	Granular
Type II	+	+	n.d.	+	Diffuse	Diffuse - granular
Type III	+	+	n.d.	n.d.	Diffuse	Diffuse
Type IV	++	++	n.d.	n.d.	Granular	Granular
Type V	++	+	Granules	++	Granular - fibrillar	Diffuse
Type VI	+	++	Granules - fibers	n.d.	Granular	Granular
Type VII	+	++	n.d.	n.d.	Diffuse	Granular
Type IX	++	++	n.d.	n.d.	Diffuse	Diffuse
Type XI	++	+	Small granules	++	Diffuse	Diffuse
Type XVII	-	-	n.d.	n.d.	-	-

Müller cells were fixed on glass chamber slides and stained with specific antibodies against types I-VII, IX, XI, and XVII collagen. LM confirmed the intracellular expression of all the above-mentioned collagens except type XVII collagen. The aspect of the collagen staining differed in aspect (diffuse, granular, or fibrillar) and intensity. In the table, the following symbols and abbreviations are used: strongly positive (++), positive (+), negative (-), not detected (n.d.), medium with 10% fetal bovine serum (FBS), and medium with 1% G5 (G5).

## Discussion

This study shows collagen synthesis by human retinal Müller cells in vitro. Müller cells expressed mRNAs coding for types I-VII, IX, and XI collagen. At the protein level, these collagens were demonstrated by immunocytochemical staining and shown to be present in the cytoplasm with LM. WB analysis of the cell extracts and of the medium in which the cells had been cultured confirmed the intracellular production and demonstrated that types I-VII, IX, and XI collagen were also secreted into the medium. The detected collagen bands could be procollagen chains as well as collagen chains, but we did not analyze this. Müller cells did not express type XVII collagen, a basement membrane protein that was recently demonstrated near photoreceptor synapses and its outer segments [25]. Apparently, Müller cells synthesize those collagens that are found in their natural vicinity (vitreous, ILL, and retina).

Although the spontaneously immortalized Müller cells have been well characterized [4] and shown to keep their main characteristics, cell models in general have as a major limitation that they are artificial in vitro systems unlike an in vivo model. Cultured Müller cells therefore might display somewhat deviant behavior because the cells are not in their natural surrounding, since they are growing on medium and moreover have shown spontaneous immortalization. To confirm our findings, primary isolated Müller cells could provide additional information, but in vivo data would be preferable.

For the WB experiments with Müller cells, ascorbic acid and β-APN were added to stimulate collagen synthesis and prevent extracellular collagen cross-linking, respectively [27,31,32]. The differences in collagen staining observed by LM—the increased extracellular staining for types I, II, V, and XI collagen paralleled by a decreased intracellular staining—were most likely the effect of ascorbic acid. The digestion experiments with collagenase confirmed the collagen nature of the bands as seen by WB.

We hypothesize that the in vitro capability of Müller cells to produce the aforementioned collagens might (1) adduce support to previously described morphological findings in the embryonic period [5,6], (2) contribute to the stable level of postnatal vitreous collagen [33], and (3) explain, in part, the origin of epiretinal membranes in pathology (see below) [7,8,34-36].

In the embryonic vitreous, the neural retina and sometimes specifically Müller cells are indicated as possible sources of vitreous and ILL collagens. In chicken embryos, retina was involved in collagen (e.g., type II) synthesis [37,38]. In the developing mouse neural retina, mRNA of types II and IX collagen has been detected [39-41]. In the human embryo, Müller cells seemed continuous with the vitreous fibrils (primarily collagen type II) present at the vitreal side [6,42,43]. The production of vitreous fibrils during embryonic growth of the eye was ascribed to Müller cells and other cell types [5,6]. Also, Müller cells were supposed to contribute to the formation of the ILL in the human embryo [6].

The postnatal vitreous has long been regarded as an almost inert extracellular matrix, in which hardly any production or breakdown of its macromolecular components occurs [20,33,44]. Recent studies question the inertness of the vitreous body and suggested turnover of vitreous components [16,18,19,45-52]. Currently, two hypotheses on vitreous aging are postulated. The first is a concept of vitreous destabilization on aging because synchisis (liquefaction) and syneresis (aggregation of vitreous matrix components) lead to the formation of spaces and aggregated collagens; the second is a view that extracellular breakdown of vitreous matrix [16] (synchisis) would coincide with production of vitreous collagen [53-55], leading to an increase in optically dense structures over time. In both views, the total amount of collagen in the human vitreous appears to remain stable during life [33], which, for the latter theory, may indicate that collagen synthesis and collagen breakdown are in equilibrium. The in vitro capacity of the Müller cell to synthesize vitreous collagens suggests a possible role in postnatal vitreous collagen synthesis. In addition, our in vitro results support the possible role of Müller cells in the formation of sublaminar vitreoretinal collagen complexes expanding on aging [11,18,19].

Müller cells are found in epiretinal membranes in pathological circumstances such as massive retinal gliosis, preretinal macular fibrosis, idiopathic epiretinal membranes, and retinal injuries or degeneration [7,8,34-36]. These membranes can contain types I-V collagen [23,24]. In morphological studies, Müller cells appear to contribute to the formation of pathological membranes [7,8,34]. However, they may not be the only type of cell involved since glial cells, astrocytes, fibrocytes, and retinal pigment epithelium have also been observed in epiretinal membranes [24,56-59].

In summary, the finding that immortalized human Müller cells synthesize collagens in vitro indicates that they might also be involved in this process in vivo. Collagen synthesis by Müller cells could explain and expand on previous morphological findings in the embryonic and postnatal period as well as in pathologic conditions. In vivo experiments will be necessary to validate our results.
